# It’s time for change: inequities and determinants of health-related quality of life among gender and sexually diverse young people in Australia

**DOI:** 10.1007/s11136-024-03633-z

**Published:** 2024-04-12

**Authors:** Sasha Bailey, Nicola Newton, Yael Perry, Lucinda Grummitt, Jeremy Goldbach, Emma Barrett

**Affiliations:** 1 The Matilda Centre for Research in Mental Health and Substance Use, Faculty of Medicine and Health, The University of Sydney, Sydney, Australia; 2 Telethon Kids Institute, University of Western Australia, Perth, Australia; 3 The Brown School, Washington University in St. Louis, St. Louis, USA

**Keywords:** Health-related quality of life, Gender, Sexual orientation, Mental health, Adolescence

## Abstract

**Purpose:**

This study was guided by three research aims: firstly, to examine the longitudinal trends of health-related quality of life (HR-QoL) among gender and sexuality diverse (LGBTQA2S+) young people through adolescence (ages 14–19); secondly, to assess longitudinal associations between poor mental health and HR-QoL among LGBTQA2S+ young people through adolescence; and thirdly, to examine differences in HR-QoL among LGBTQA2S+ young people during early adolescence (ages 14 and 15) depending on select school-, peer-, and parent-level factors.

**Methods:**

This study used three of nine available waves of data from a large population-level, probability sample-based, longitudinal cohort study, namely the K′ cohort: children aged 4–5 years old at time of study enrolment followed-up biennially (~ 61% retention rate). HR-QoL weighted means and standard deviations were calculated using Child Health Utility 9D (CHU-9D) scores for LGBTQA2S+ participants at ages 14 and 15 (Wave 6), ages 16 and 17 (Wave 7), and ages 18 and 19 (Wave 8). To strategically identify policy-relevant modifiable behavioural factors suitable for prevention and early intervention, non-parametric Wilcoxon signed-rank tests tested differences in mean CHU-9D ranks at ages 14 and 15 (Wave 6) between groups (gender identity: trans vs. cis; identity-level sexuality: gay, lesbian, bisexual, or other diverse sexuality vs. heterosexual; group-level sexuality: sexually diverse vs. not sexually diverse) and selected school factors (school acceptance, belonging, freedom of expression), peer factors (peer relationship quality, trust, respect), and family factors (parental acceptance, understanding, trust), with Hedge’s *g* correction statistics computed for effect sizes. Longitudinal associations between gender, sexuality, and poor mental health (depressive symptoms, anxiety, symptoms, self-harm thoughts/behaviour, and suicidal thoughts/behaviour) and HR-QoL were tested using mixed-effects models with random intercepts and random slopes for nested clustering (participants within postcodes).

**Results:**

HR-QoL disparities disproportionately affecting LGBTQA2S+ groups relative to their cisgender, heterosexual peers, were well-established by age 14 to 15 relatively steeper reductions in HR-QoL were observed throughout adolescence among all LGBTQA2S+ groups, with HR-QoL widening the most for trans participants. Poor mental health was significantly associated with HR-QoL declines. LGBTQA2S+ participants with positive school- and parent factors related to feelings of acceptance, belonging, and freedom of self-expression, reported significantly higher HR-QoL during early adolescence.

**Conclusion:**

Evidence-based public health policy responses are required to address the dire HR-QoL inequities among LGBTQA2S+ young people, particularly trans young people. Prioritising the promotion of school- and family-based interventions which foster LGBTQA2S+ inclusivity, acceptance, and a sense of belonging from early adolescence through young adulthood, represents a feasible, evidence-based, and cost-effective response to address these HR-QoL disparities

## Introduction

Gender and sexually diverse young people (including those who describe aspects of their identities with terms such as lesbian, gay, bisexual, trans, nonbinary, queer, questioning, asexual, agender, two-spirit, or who otherwise have experiences of queerness and/or transness and are not cisgender—have a gender identity congruent with the gender presumed for them at birth—or heterosexual—have sexual and/or romantic attraction exclusively for those of ‘the other’ gender’; henceforth respectfully referred to using the corresponding acronym and umbrella term ‘LGBTQA2S+’ young people) derive euphoria, joy, and vitality from experiences shaped by their gender and sexuality [1]. Notwithstanding, there is growing awareness that they face some of the largest health inequities documented in the twenty-first century [1]. Evidence from Australia [2–4] and abroad [5–8] has consistently highlighted the alarmingly high rates of poor mental health experienced by many LGBTQA2S+ young people, largely attributable to chronic stigma and discrimination [9–11] associated with societal norms and expectations of cisnormativity and heterosexism [12, 13].

Recent years have seen an exponential increase in research regarding health-related quality of life (HR-QoL) [14], a multidimensional concept of the perceived physical, mental, emotional, and social dimensions of health and well-being [15]. HR-QoL measures the impacts of health status on quality of life [16] and thus, is a valuable tool for policymakers to broadly consider health inequities and progress of society [17, 18]. Given the recent promulgation of government policy documents articulating health strategy and planning activities related to the health of LGBTQA2S+ young people in Australia [1, 19, 20], population-based, nationally representative evidence regarding trends and underlying causes of HR-QoL among LGBTQA2S+ young people is urgently required to inform these policy priorities.

There is a paucity of Australian research, however, investigating HR-QoL among LGBTQA2S+ young people. Much of the literature stems from international samples [21, 22]. A recent study assessed quality of life in a clinical sample of 525 trans children and adolescents aged 6 to 17 years presenting to a gender service in Melbourne, Australia, compared with age-matched cisgender peers in the Australian general population [23]. This study found that quality of life scores were significantly lower among trans people compared with their cisgender peers and particularly worse among those reporting poor mental health [23]. Previous research has also examined population-level norms of HR-QoL among young people in the general population and the associated burden of poor mental health [24]; however, gender and sexuality-based differences were not explored. Other valuable studies have focused on LGBTQA2S+ adults [25–27] or were cross-sectional [28], thus precluding conclusions regarding HR-QoL during the developmentally critical years of adolescence for LGBTQA2S+ young people. Indeed, school-, peer-, and family-level factors are largely neglected in HR-QoL studies of LGBTQA2S+ young people despite overwhelming evidence suggesting these three contexts are among the most effective for promoting their mental health and general well-being [29–31].

To begin to fill these gaps in the existing literature, the present study aimed to (a) quantify and examine the longitudinal trends of HR-QoL among LGBTQA2S+ young people through adolescence (ages 14–19 years); (b) assess the longitudinal associations between poor mental health and HR-QoL among LGBTQA2S+ young people through adolescence; and (c) examine differences in HR-QoL among LGBTQA2S+ young people during early adolescence (ages 14 and 15) depending on selected school-, peer-, and parent-level factors. The researchers decided to examine these factors at ages 14 and 15 because producing a snapshot of modifiable determinants relevant to the HR-QoL among LGBTQA2S+ young people during early adolescence would produce insights into prevention and early intervention targets to potentially avert and reduce HR-QoL inequities that exacerbate through adolescence.

## Methods

### Study design and participants

The present study used data drawn from the Longitudinal Study of Australian Children (LSAC), a population-level (probability sample), longitudinal, dual, cross-sequential cohort study, namely the ‘K’ cohort: children aged 4–5 years old at time of study enrolment) [32]. The LSAC sample was selected from the Medicare Australia enrolments database, Australia’s most comprehensive population database, particularly of young children [32]. A two-stage clustered design was employed wherein 311 geographic postcodes were randomly selected, following which, children were subsequently randomly selected within each postcode [32]. Stratification was utilised to ensure that numbers of children selected were roughly proportionate to the total number of children within each Australian state/territory, capital city districts and broader regional surrounding areas [32]. This method of accounting for the number of children in each postcode meant that potentially participants across Australia had an approximately equal chance of selection (approximately one in 25) [32]. This study used data from Wave 6 (ages 14 and 15), Wave 7 (ages 16 and 17), and Wave 8 (ages 18 and 19) of the K cohort, who were born between March 1999 and February 2000 and were followed up in 2018 when they were 18 or 19 years old. The response rates were 82% for Wave 6; 76% for Wave 7; and 77% for Wave 8. At Wave 8, 61% of the Wave 1 sample had been retained [32]. Participants were visited once every two years, whereby interviews, direct observations, and assessments were conducted, with total time taken to complete the assessment protocol approximating on average 60 min per participant per wave. Additional detail, including the use of nonprobability-based selection of participants via geographically representative postcode sampling, is published elsewhere [33]. Participants consented verbally to each LSAC wave of data collection in accordance with ethical standards outlined in the National Statement on Ethical Conduct in Research Involving Humans. All materials and survey content included in LSAC have received ethical review and approval by the Australian Institute of Family Studies Ethics Committee, a National Health and Medical Research Council-registered Human Research Ethics Committee.

### Measures

#### Gender identity

Participants were asked about their sex assigned at birth (male or female) and current gender identity (male; female; transgender, male to female; transgender, female to male; genderqueer; and other). Trans people were participants who explicitly identified as transgender or indicated a current gender different from their assigned sex. Participants with concordant sex and gender responses were classified as cisgender.

#### Sexuality

Participants were asked, “Which of the following categories best describes how you think of yourself?” Response options were “heterosexual or straight,” “gay or lesbian,” “bisexual,” “other,” and “don’t know.”

A “sexuality diversity” variable was also computed, with a group including all people who shared that they were sexually diverse on at least one of three items relating to sexual identity, attraction, and behaviour. Specifically, this group-level variable included (a) those who identified as gay, lesbian, bisexual, other, or don’t know in response to the identity question; (b) male participants who were equally, mostly, or only attracted to men; female participants who were equally, mostly, or only attracted to women; and people who were unsure of their sexual attraction or had never felt sexual attraction in response to the sexual attraction item; and (c) male participants who had had sex with men or both men and women; and female participants who had had sex with women or both women and men in response to the sexual behaviour item. Those who did not meet these criteria were coded as not sexually diverse. Whereas the sexuality identity variables focused on the identity labels participants used to describe themselves (e.g., gay, lesbian), the sexuality diversity group variable drew from this identity variable and participants’ responses regarding their sexual attraction and behaviour. Hence, this sexuality group variable ensured representation of participants who may describe themselves as heterosexual but, for example, also report same-gender attraction or sexual relations. This methodology has been used in previous research examining mental health disparities in a United Kingdom-based sample of sexually diverse adolescents [34], which suggested sexuality-based disparities in mental health persist regardless of whether young people describe their identities as such.

#### HR-QoL

The present study used the Child Health Utility-9D (CHU-9D) scale [35], a measure of HR-QoL originally developed for children aged 7 to 11 years but that has since been validated in national Australian and global samples of adolescents aged 11 to 17 years [36–38]. The CHU-9D features nine questions with five response levels per question, with mean response scores of 1 representing full health, 0 representing death, and less than 0 representing worse off than death [36]. The CHU-9D questionnaire was only administered to participants during LSAC Waves 6, 7, and 8.

#### Mental health

The 13-item Short Mood and Feelings Questionnaire [39], a 13-item self-report checklist of core depression symptoms validated for use among children and adolescents, was used to assess depressive symptomatology during the past 2 weeks. It features 13 statements such as “I felt nobody loved me”; participants responded on a 3-point scale (0 = *not true*, 1 = *sometimes true*, 2 = *true*). Participants’ responses were summed (range 0–26), and scores of 8 or higher were defined as indicative of probable depressive disorder [40]. The questionnaire has been previously validated as a sound self-report depression checklist of core symptoms for children and adolescents aged 8 to 16 [41]. It was included in LSAC Waves 6 and 7.

The 8-item version of the Spence Children’s Anxiety Scale [42] was used to assess the frequency of anxiety symptomatology among participants. Example items are “I am scared of the day” and “I worry about things.” Participants responded on a scale ranging from 0 (*never*) to 3 (*always*). Response scores were summed for a total continuous measure of anxiety (range: 0–24), with higher scores indicated higher levels of anxiety. Although the full scale consists of 44 items, the 8-item version has been found to have good internal consistency and convergent and diversity validity with samples of young people up to age 17 [43]. The scale was included in LSAC Waves 6 and 7.

Participants were asked to self-report (yes or no) whether in the past 12 months they had experienced self-harm ideation (i.e., “thought about hurting yourself on purpose in any way”) and self-harm behaviours (i.e., “hurt yourself on purpose in any way”). Self-harm ideation and behaviours were assessed in LSAC Waves 6, 7, and 8 (age 18/19).

Participants were asked to self-report (yes or no) whether in the past 12 months they had experienced suicidal ideation (i.e., “ever seriously consider attempting suicide”) and suicidal planning (i.e., “make a plan about how you would attempt suicide”). Additionally, participants were also asked to respond on a scale from 0 (*0 times*) to 4 (*6 or more times*) how often during the past 12 months they had attempted suicide. Participants who responded 1 or more times were coded as experiencing suicide attempt in the past 12 months. Suicidal ideation, planning, and attempt were assessed in LSAC Waves 6, 7, and 8.

#### School factors

Since Wave 5 of LSAC, 12 of the original 18 items from the Psychological Sense of School Membership (PSSM) scale have been used to measure participants’ perceived sense of school belonging. The PSSM was developed and validated as a measure of adolescents’ perceived belonging or psychological membership in the school environment. Previous population-based studies of children and adolescents in Australia have demonstrated sound reliability and validity of the PSSM scale. The 12 items included in LSAC feature positive and negative statements (e.g., “I can be myself at school” or “Sometimes I don’t feel as if I belong here”), with responses ranging from 1 (*not at all true*) to 5 (*completely true*). Sum scores range from 12 to 60 (negative items were reverse scored). Higher PSSM scores denote more positive youth perceptions of school belonging. Four PSSM items (“It is hard for people like me to be accepted here,” “Sometimes I don’t feel as if I belong here,” “I can really be myself at school,” and “I wish I were at a different school”) were extracted for the present study (with the other eight PSSM items excluded) because past empirical and theoretical research has highlighted the pivotal role of schools in fostering a sense of inclusion and acceptance of LGBTQA2S+ students, encouraging them to be themselves at school. When that is not possible, LGBTQA2S+ young people may wish to be in a different school that offers this inclusive, safe environment. This approach of singling out universal school climate items due to their unique implications for LGBTQA2S+ inclusivity and support has been used before [44]. At LSAC Wave 6, these four PSSM items produced excellence internal consistency (α = 0.95 for all).

#### Peer factors

Characteristics of participants’ peer relationships were drawn from the peer attachment subscale of the Inventory of Peer and Parental Attachment [45]. Participants considered statements regarding characteristics of their peer relationship (e.g., “I trust my friends”) and responded on a 5-point scale to indicate how true these statements were for them (1 = *almost always true*, 5 = *almost never true*). Of the inventory’s eight items, three were included in the current study (“I feel my friends are good friends,” “I trust my friends,” and “My friends respect my feelings”), because these interpersonal attributes were deemed especially pertinent to fostering peer inclusion and support for gender and sexuality diversity. These items were dichotomised (0 = *no*, 1 = *yes*), with participants who responded “sometimes true,” “seldom true,” or “almost never true” coded as 0 and those responding “almost always true” or “often true” coded as 1. This approach of dichotomising participants with and without these peer-level factors has been used in previous analyses of LSAC data [46]. Within this study at LSAC Wave 6, all peer-related items indicated excellent internal consistency (α ≥ 0.97 for all).

#### Family factors

Since Wave 4 of LSAC, eight items drawn from the trust and communication subscale of the People In My Life measure [47] have been used. The measure has been validated among children aged 8 to 12 years and adolescents aged 13 to 18 years [48]. Of these eight items, three were used in the current study: “My parents accept me as I am,” “My parents understand me,” and “I trust my parents.” Participants responded on a 4-point scale (1 = *almost never or never true* to 4 = *almost always or always true*). These three items were chosen given their articulation of elements of trust, acceptance, and understanding between participants and their parents—key aspects of parent–child relationships found to be affirming, supportive, and inclusive of gender and sexuality diversity in several previous studies [29, 30]. This approach of hand-picking individual items from universal measures has been used in examining school-level determinants of LGBTQA2S+ youth mental health [44]. These three family-level items were dichotomised, with participants responding “almost never or never true” or “sometimes true” as 0 (*no*; parental factor absent) and “often true” or “almost always or always true” as 1 (*yes*; parental factor present). At Wave 6, all parent items indicated excellent internal consistency (*α* ≥ 0.93 for all).

### Statistical analyses

**Fig. 1:**
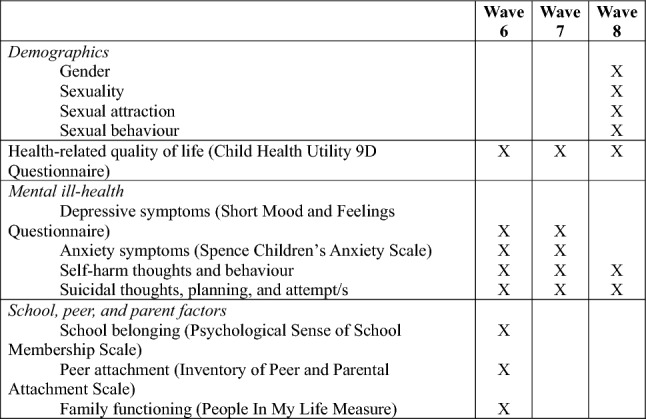
Key variables from relevant LSAC waves used for the present study

For the present study, only data from LSAC Waves 6, 7, and 8 was used because these are the only waves where information regarding HR-QoL, self-harm, and suicidality data was collected. Figure 1 depicts the key variables and corresponding wave of data collection used for the present analysis.

To track HR-QoL through adolescence among LGBTQA2S+ participants, HR-QoL weighted means and standard deviations were calculated using CHU-9D scores for LGBTQA2S+ participants at ages 14 and 15 (Wave 6), ages 16 and 17 (Wave 7), and ages 18 and 19 (Wave 8) using wave-specific composite weights to align estimates with nationally representative sample characteristics yielded at baseline. Nonparametric Wilcoxon signed-rank tests were used to test compare mean CHU-9D scores between groups (gender identity: trans vs. cis; sexuality identity: gay, lesbian, bisexual, or other diverse sexuality vs. heterosexual; sexuality group: sexually diverse vs. not sexually diverse). Hedge’s *g* correction statistics were computed to estimate effect sizes associated with differences (small: *d* ≥ 0.20; medium: *d* ≥ 0.50; large: *d* ≥ 0.80). Effect sizes below .20 are considered negligible.

To test longitudinal associations between gender or sexuality and HR-QoL across the three timepoints (Waves 5, 6, and 8), hierarchical linear mixed-effects regression models with random intercepts and random slopes were computed. To account for within-subject error associated with repeated measures, a random intercept of participant record IDs was included to allow the intercept to vary by participant. Furthermore, introduction of random slopes allowed for nested random effects to account for participant clustering in geographical postcodes. Time (i.e., the three LSAC waves) was transformed into a categorical variable and included as a covariate in all mixed-effects models. Beta coefficients and 95% confidence intervals were inspected and a statistical significance threshold of *p* < .05 was used.

Differences in HR-QoL associated with school-, peer-, and parent factors were calculated and tests using the same process were used to calculate weighted means and standard deviations of CHU-9D scores for LGBTQA2S+ participants with and without each factor, including Wilcoxon signed-rank tests and Hedge’s *g* statistics. These specific school, peer, and parent factors were strategically selected as designations of policy-relevant modifiable behavioural factors suitable for prevention and early intervention to promote HR-QoL and mental health among LGBTQA2S+ young people.

All statistical analyses were conducted in the statistical package R Studio, version 4.2.2.1. This paper was prepared in accordance with STROBE guidelines for observational cohort studies (see https://www.strobe-statement.org).

## Results

In total, 3127 participants completed Wave 8 follow-up, along whom, 36 participants were trans and 2619 were cisgender. Regarding sexual identity, 56 participants identified as gay, 225 as bisexual, and 39 as having another diverse sexuality (henceforth referred to as people with other diverse sexualities). At a group level, 402 participants were sexually diverse and 2211 participants were not sexually diverse.

### Characteristics of the sample

Among the present sample of 3127 participants at baseline (LSAC Wave 6), the mean age was 14.3 (SD = 0.47). At baseline, 1606 (51.4%) participants had a male binary sex presumed at birth, and 1521 (48.6%) participants had a female binary sex presumed at birth. Moreover, 1865 (59.8%) participants lived in a metropolitan area and 1255 (40.2%) participants lived in a non-metropolitan area.

Additional demographic characteristics of participants by gender and sexuality are detailed in Table 1.

**Table 1:** Demographic characteristics of K^a^ cohort participants by gender and sexuality at Wave 6 (ages 14 to 15 years)^b^

	Cis	Trans	Heterosexual	Gay/Lesbian	Bisexual	Other	Non-sexuality diverse	Sexuality diverse
All (*N*^c^)	2619	36	2261	56	225	39	2211	402
Age—M^d^ (SD^e^)	14.4 (0.49)	14.3 (0.48)	14.4 (0.49)	14.4 (0.50)	14.1 (0.49)	14.3 (0.48)	14.4 (0.49)	14.4 (0.49)
Binary sex presumed at birth								
Female—*N* (%)	1276 (49.8)	20 (55.6)	1041 (47.0)	24 (43.6)	152 (69.4)	25 (69.4)	1016 (46.9)	226 (63.0)
Male—*N* (%)	1287 (50.2)	16 (44.4)	1174 (53.0)	31 (56.36)	67 (30.6)	11 (30.6)	1150 (53.1)	133 (37.0)
Australian state of residence—*N*								
New South Wales	785	15	677	19	75	11	662	120
Victoria	562	*	482	11	45	*	470	78
Queensland	497	*	434	*	43	*	425	68
South Australia	164	*	146	*	12	*	142	20
Western Australia	267	*	228	*	24	*	224	38
Tasmania	92	*	79	*	*	*	78	14
Northern Territory	26	*	22	*	*	*	22	*
Australian Capital Territory	69	*	61	*	*	*	61	*
Region of residence								
Non-metropolitan	957 (39.0)	13 (36.1)	836 (39.4)	20 (38.5)	72 (34.1)	11 (31.4)	819 (39.4)	120 (35.0)
Metropolitan	1497 (61.0)	23 (63.9)	1286 (60.6)	32 (61.5)	139 (65.9)	24 (68.6)	1258 (60.6)	223 (65.0)
Index of Relative Socio-economic Disadvantage Scores—M (SD)	1017 (65.4)	1008 (62.9)	1019 (64.0)	991 (75.4)	1004 (70.8)	1036 (59.9)	1019 (64.1)	1005 (69.5)

^a^The Longitudinal Study of Australian Children (LSAC) comprises two cohorts, an older (‘K’) cohort, and younger (‘B’) cohort. The K cohort were born between March 1999 and February 2000

^b^Gender and sexuality indicators were only included in the LSAC dataset at Wave 8 (ages 18 to 19 years). The application of gender and sexuality indicators to retrospectively identify LGBTQA+ participants in longitudinal observational cohort studies are ethically permissible and epidemiologically rigorous where gender and sexuality is inconsistently recorded throughout follow-up studies given the evolutionary nature of the development of gender and sexuality. This approach ensures optimal coverage of people who have shared information about their gender or sexuality during the study period. This rationale is formally detailed in a manuscript currently under peer-review with a journal specialising in trans health

^c^Count

^d^Mean

^e^Standard deviation

*The LSAC dataset is governed by Australian Institute of Family Studies rules which require the censoring of values ≤ 10

### Tracking disparities in HR-QoL among LGBTQA2S+ young people through adolescence

#### Gender identity

As shown in Table 2, at ages 14 to 19, trans participants reported significantly lower HR-QoL relative to cisgender participants (*p* < .01 for all age categories). The difference in HR-QoL between trans participants (*M* = 0.50, *SD* = 0.09) vs. cisgender participants (*M* = 0.71, *SD* = 0.05) at ages 18 and 19 yielded a large effect size (*g* = .91).

**Table 2:** Weighted mean health-related quality of life scores among gender and sexuality diverse young people by across LSAC waves 6–8

	Gender identity				Sexuality (identity-level)										Sexuality (group-level)			
	Trans	Cis	*p*	Hedges g 95% CI	Gay/Lesbian	*p*	Hedges g 95% CI	Bisexual	*p*	Hedges g 95% CI	Other diverse Sexuality	*p*	Hedges g 95% CI	Heterosexual	Sexuality Diverse	Not Sexuality Diverse	*p*	Hedges g 95% CI
	M (SD)	M (SD)			M (SD)			M (SD)			M (SD)			M (SD)	M (SD)	M (SD)		
Wave 6 (age 14/15) 2013–2014	0.71 (0.07)	0.79 (0.04)	*0.004*	0.11 − 0.23, 0.46	0.72 (0.05)	*< 0.001*	− 0.79 − 1.06, − 0.51	0.69 (0.06)	*<0.001*	− 0.67 − 0.81, − 0.52	0.69 (0.05)	*0.006*	− 0.94 − 1.31, − 0.57	0.81 (0.04)	0.70 (0.06)	0.81 (0.04)	*< 0.001*	0.12 0.00, 0.24
Wave 7 (age 16/17) 2015–2016	0.65 (0.1)	0.77 (0.05)	*0.0012*	− 0.72 − 1.05, − 0.38	0.70 (0.05)	*< 0.001*	− 0.65 − 0.94, − 0.37	0.65 (0.07)	*< 0.001*	− 0.69 − 0.84, − 0.55	0.58 (0.08)	*0.0011*	− 0.78 − 1.16, − 0.41	0.79 (0.04)	0.67 (0.07)	0.79 (0.04)	*< 0.001*	− 0.69 − 0.82, − 0.56
Wave 8 (age 18/19) 2017–2018	0.50 (0.09)	0.71 (0.05)	*< 0.001*	0.91 − 1.24, − 0.57	0.57 (0.05)	*< 0.001*	− 0.79 − 1.06, − 0.51	0.57 (0.06)	*< 0.001*	− 0.67 − 0.81, − 0.52	0.48 (0.06)	*< 0.001*	− 0.94 − 1.31, − 0.57	0.72 (0.05)	0.58 (0.07)	0.72 (0.05)	*< 0.001*	− 0.71 − 0.84, − 0.59

*p* < 0.05 indicates moderate statistically significant results, *p* < 0.01 indicates strong evidence of statistically significant results, *p* < 0.001 indicates very strong evidence of statistically significant results

#### Sexuality

As shown in Table 2, across all timepoints (ages 14 and 15, ages 16 and 17, and ages 18 and 19), gay and lesbian adolescents reported significantly lower HR-QoL scores compared to their heterosexual peers, all producing medium effect sizes (*g* = − 0.79 to -.65; *p* < .001 for all).

Bisexual participants also reported significantly lower HR-QoL scores across all three timepoints compared with their heterosexual peers, all associated with medium effect sizes (*g* = − 0.69 to − 0.71).

Participants with other diverse sexualities consistently reported lower HR-QoL scores at all age groups relative to their heterosexual peers (*p* < .01 for all). At ages 14 and 15 and ages 18 and 19, differences in HR-QoL disproportionately affected participants with other diverse sexualities compared with their heterosexual peers, associated with a large effect size (*g* = 0.94 for both).

At a group level, sexually diverse participants reported lower HR-QoL scores across all three timepoints through adolescence, all were associated with medium effect sizes (*g* = − 0.71 to 0.12; *p* < .001 for all).

### Longitudinal disparities in HR-QoL

Results from mixed-effects modelling with a random intercept using participants’ IDs nested in a random slope using participants postcodes were used to test longitudinal gender and sexuality disparities in health-related quality of life.

#### Gender identity

Findings revealed that trans young people experienced significantly lower HR-QoL scores through adolescence compared with their cisgender peers (*β* = − 0.42, 95% CI [− 0.64, − 0.20], *p* < .001).

#### Sexuality

Bisexual participants (*β* = − 0.12, 95% CI [− 0.12, − 0.03], *p* = .01) and participants with other diverse sexualities (*β* = − 0.36, 95% CI [− 0.58, − 0.13[*p* < .001) reported significantly lower HR-QoL scores through adolescence compared with their heterosexual counterparts. Gay and lesbian participants did not report significantly different HR-QoL scores over time compared with their heterosexual peers.

At a group level, sexually diverse participants reported significantly lower HR-QoL scores compared with their nonsexually diverse peers (*β* = − 0.18, 95% CI [− 0.25, − 0.10], *p* < .001).

### Longitudinal impacts of poor mental health on HR-QoL among LGBTQA2S+ young people through adolescence

Multilevel linear mixed-effects models with a random intercept nested in geographical postcodes were computed to test longitudinal associations of repeated measures of poor mental health study factors (depressive symptoms, anxiety symptoms, past-12-month self-harm ideation and attempt, and past-12-month suicidal ideation, planning, and attempt) on HR-QoL outcomes (CHU-9D scores) among each LGBTQA2S+ subgroup. Significant results are described.

#### Gender identity

Among trans participants, depressive symptoms (*β* = − 0.06, 95% CI [− 0.09, − 0.02]), past-12-month self-harm ideation (*β* = − 0.18, 95% CI [− 0.29, − 0.08]), past-12-month suicidal ideation (*β* = − 0.21, 95% CI [− 0.31, − 0.12]), and past-12-month suicidal planning (*β* = − 0.13, 95% CI [− 0.23, − 0.03]) were significantly associated with reduced HR-QoL.

#### Sexuality

Depressive symptoms (*β* = − 0.02, 95% CI [− 0.02, − 0.01]), past-12-month self-harm ideation (*β* = − 0.16, 95% CI [− 0.23, − 0.09]), past-12-month self-harm attempt (*β* = − 0.16, 95% CI [− 0.24, − 0.08]), past-12-month suicidal ideation (*β* = − 0.20, 95% CI [− 0.28, − 0.12]), and past-12-month suicidal planning (*β* = − 0.17, 95% CI [− 0.26, − 0.08]) were significantly associated with reduced HR-QoL among gay and lesbian participants.

Among bisexual participants, depressive symptoms (*β* = − 0.02, 95% CI [− 0.03, − 0.02]), past-12-month self-harm ideation (*β* = − 0.21, 95% CI [− 0.24, − 0.17]), past-12-month self-harm attempt (*β* = − 0.18, 95% CI [− 0.23, − 0.14]), past-12-month suicidal ideation (*β* = − 0.16, 95% CI [− 0.20, − 0.12]), past-12-month suicidal planning (*β* = − 0.19, 95% CI [− 0.23, − 0.14]), and past-12-month suicide attempt (*β* = − 0.08, 95% CI [− 0.12, − 0.04]) were significantly associated with reduced HR-QoL scores.

Past-12-month self-harm ideation (*β* = − 0.23, 95% CI [− 0.23, − 0.13]), past-12-month self-harm attempt (*β* = − 0.15, 95% CI [− 0.26, − 0.04]), past-12-month suicidal ideation (*β* = − 0.25, 95% CI [− 0.35, − 0.16]), and past-12-month suicidal planning (*β* = − 0.17, 95% CI [− 0.29, − 0.04]) were significantly associated with lower HR-QoL scores among participants with other diverse sexualities.

At a group level, sexually diverse people who experienced depressive symptoms (*β* = − 0.02, 95% CI: [− 0.03, − 0.02]), past-12-month self-harm ideation (*β* = − 0.21, 95% CI [− 0.24, − 0.18]), past-12-month self-harm attempt (*β* = − 0.19, 95% CI [− 0.22, − 0.16]), past-12-month suicidal ideation (*β* = − 0.18, 95% CI [− 0.22, − 0.15]), past-12-month suicidal planning (*β* = − 0.18, 95% CI [− 0.21, − 0.14]), and past-12-month suicide attempt (*β* = − 0.07, 95% CI [− 0.10, − 0.03]) reported significantly lower HR-QoL scores compared with those who did not report experiencing these poor mental health outcomes (Tables 3).

**Table 3:** Longitudinal associations between mental ill-health and quality of life scores among gender and sexuality diverse young people through ages 14-19 (2013-2018)

	Trans^a^		Gay/Lesbian^b^		Bisexual^b^		Other diverse sexuality^b^		Sexuality diverse^b^	
	B SE	95% CI *p*	B SE	95% CI *p*	B SE	95% CI *p*	B SE	95% CI *p*	B SE	95% CI *p*
Depressive symptoms	− 0.06 0.02	− 0.09, − 0.02 *< 0.001*	− 0.02 0.00*	− 0.02, − 0.01 *< 0.001*	− 0.02 < 0.00	− 0.03, − 0.02 *< 0.001*	− 0.02 0.02	− 0.07, 0.03 *0.45*	− 0.02 < 0.00	− 0.03, − 0.02 *< 0.001*
Anxiety symptoms	0.02 0.04	− 0.07, 0.10 *0.69*	− 0.00 0.01	− 0.02, 0.01 *0.46*	− 0.00 0.01	− 0.02, 0.01 *0.62*	0.03 0.04	− 0.05, 0.12 *0.43*	0.02 0.01	− 0.00, 0.04 *0.07*
Past 12-month self-harm ideation	− 0.18 0.05	− 0.29, − 0.08 *< 0.001*	− 0.16 0.04	− 0.23, − 0.09 *< 0.001*	− 0.21 0.02	− 0.24, − 0.17 *< 0.001*	− 0.23 0.05	− 0.32, − 0.13 *< 0.001*	− 0.21 0.01	− 0.24, − 0.18 *< 0.001*
Past 12-month self-harm attempt/s	− 0.11 0.06	− 0.21, 0.00 *0.06*	− 0.16 0.04	− 0.24, − 0.08 *< 0.001*	− 0.18 0.02	− 0.23, − 0.14 *< 0.001*	− 0.15 0.06	− 0.26, − 0.04 *0.01*	− 0.19 0.02	− 0.22, − 0.16 *< 0.001*
Past 12-month suicidal ideation	− 0.21 0.05	− 0.31, − 0.12 *< 0.001*	− 0.20 0.04	− 0.28, − 0.12 *< 0.001*	− 0.16 0.02	− 0.20, − 0.12 *< 0.001*	− 0.25 0.05	− 0.35, − 0.16 *< 0.001*	− 0.18 0.02	− 0.22, − 0.15 *< 0.001*
Past 12-month suicidal planning	− 0.13 0.05	− 0.23, − 0.03 *0.01*	− 0.17 0.05	− 0.26, − 0.08 *< 0.001*	− 0.19 0.02	*− 0.23, − 0.14* *< 0.001*	− 0.17 0.06	− 0.29, − 0.04 *0.01*	− 0.18 0.02	− 0.21, − 0.14 *< 0.001*
Past 12-month suicide attempts	− 0.00 0.03	− 0.07, 0.06 *0.90*	− 0.06 0.04	− 0.13, 0.02 *0.14*	− 0.08 0.02	− 0.12, − 0.04 *< 0.001*	− 0.05 0.04	− 0.13, 0.04 *0.29*	− 0.07 0.02	− 0.10, − 0.03 *< 0.001*

^a^Reference group comprised cisgender participants

^b^Reference group comprised heterosexual participants

*p* < 0.05 indicates moderate statistically significant results, *p* < 0.01 indicates strong evidence of statistically significant results, *p* < 0.001 indicates very strong evidence of statistically significant results

### School-, peer-, and family-related determinants of HR-QoL among LGBTQA2S+ young people at age 14 and 15

#### Gender identity

As shown in Table 4, among trans participants, those who indicated that their parents understand them reported significantly higher HR-QoL scores compared with those who did not, and this was associated with a large effect size (*g* = 1.01, *p* < .05). No other statistically significant differences were detected.

**Table 4:** Weighted mean health-related quality of life scores among gender and sexuality diverse young people at age 14-15 with school-level risk and protective factors

		Trans			Gay/Lesbian			Bisexual			Other Diverse Sexuality			Sexuality diverse		
		M (SD)	*p*	Hedge’s *g* 95% CI	M (SD)	*p*	Hedge’s *g* 95% CI	M (SD)	*p*	Hedge’s *g* 95% CI	M (SD)	*p*	Hedge’s *g* 95% CI	M (SD)	*p*	Hedge’s *g* 95% CI
School-level factors																
I find it hard to be accepted	Yes	0.58 (0.12)	*0.27*	− 0.54 − 1.48, 0.40	0.63 (0.08)	*0.37*	− 0.43 − 1.18, 0.32	0.6 (0.08)	*0.006*	− 0.56 − 0.94, − 0.18	0.55 (0.1)	*0.338*	− 0.71 − 1.54, 0.14	0.59 (0.08)	0.0013	− 0.55 − 0.87, − 0.24
	No	0.73 (0.06)			0.74 (0.04)			0.7 (0.06)			0.74 (0.03)			0.72 (0.05)		
I feel I don’t belong	Yes	0.58 (0.09)	*0.14*	− 0.55 − 1.31, 0.22	0.57 (0.05)	*0.019*	− 0.83 − 1.52, − 0.12	0.54 (0.07)	*< 0.001*	− 0.98 − 1.29, − 0.67	0.64 (0.007)	*0.52*	− 0.31 − 1.04, 0.43	0.56 (0.07)	*< 0.001*	− 0.89 − 1.15, − 0.62
	No	0.75 (0.06)			0.76 (0.04)			0.75 (0.05)			0.71 (0.04)			0.75 (0.05)		
I can be myself at school	Yes	0.77 (0.05)	*0.08*	0.70 − 0.02, 1.42	0.76 (0.04)	*< 0.001*	0.58 − 0.01, 1.18	0.74 (0.05)	*< 0.001*	0.74 0.44, 1.05	0.75 (0.03)	*0.37*	0.53 − 0.18, 1.24	0.75 (0.05)	*< 0.001*	0.69 0.44, 0.95
	No	0.6 (0.1)			0.62 (0.05)			0.56 (0.06)			0.59 (0.07)			0.58 (0.06)		
I wish I was at a different school	Yes	0.55 (0.12)	*0.14*	− 0.72 − 1.59, 0.17	0.43 (0.04)	*0.06*	− 1.45 − 2.62, − 0.26	0.58 (0.07)	*0.01*	− 0.44 − 0.80, − 0.07	0.45 (0.04)	*0.07*	− 1.16 − 2.21, − 0.09	0.57 (0.07)	*0.001*	− 0.58 − 0.91, − 0.25
	No	0.74 (0.06)			0.74 (0.04)			0.71 (0.06)			0.73 (0.04)			0.72 (0.05)		
Peer-level factors																
I feel my friends are good friends	Yes	0.73 (0.06	*0.38*	0.57 − 0.47, 1.59	0.73 (0.05)	*0.78*	− 0.85, 0.83	0.70 (0.06)	*0.006*	0.63 0.21, 1.05	0.69 (0.04)	*0.96*	0.12 − 0.81, 1.05	0.71 (0.06)	*0.006*	0.46 0.11, 0.81
	No	0.59 0.18			0.70 (0.02)			0.56 (0.07)			0.71 (0.11)			0.60 (0.07)		
I feel I can trust my friends	Yes	0.76 (0.06)	*0.06*	0.94 0.08, 1.79	0.73 (0.04)	*0.73*	0.14 − 0.54, 0.82	0.70 (0.06)	*0.03*	0.41 0.06, 0.76	0.73 (0.03)	*0.35*	0.45 − 0.27, 1.17	0.71 (0.06)	*0.02*	0.36 0.07, 0.64
	No	0.46 (0.08)			0.69 (0.05)			0.62 (0.06)			0.63 (0.07)			0.63 (0.06)		
I feel my friends respect my feelings	Yes	0.77 (0.05)	*0.07*	0.85 0.04, 1.66	0.75 (0.04)	*0.70*	0.79 0.03, 1.55	0.69 (0.06)	*0.30*	0.30 − 0.08, 0.67	0.71 (0.05)	*0.30*	0.42 − 0.32, 1.15	0.71 (0.06)	*0.02*	0.38 0.07, 0.68
	No	0.5 (0.09)			0.61 (0.07)			0.67 (0.06)			0.65 (0.06)			0.66 (0.06)		
Parent-level factors																
Parents accept me	Yes	0.77 (0.05)	*0.06*	1.14 0.22, 2.04	0.76 (0.04)	*0.006*	1.41 0.57, 2.23	0.73 (0.05)	*0.0014*	0.65 0.30, 1.00	0.71 (0.04)	*0.6279*	0.61 − 0.43, 1.64	0.74 (0.05)	*< 0.001*	0.76 0.45, 1.06
	No	0.38 (0.09)			0.49 (0.04)			0.56 (0.09)			0.53 (0.18)			0.56 (0.08)		
Parents understand me	Yes	0.77 (0.06)	*0.04*	1.01 0.14, 1.85	0.76 (0.04)	*0.05*	0.83 0.16, 1.48	0.73 (0.05)	*< 0.001*	0.68 0.37, 0.98	0.72 (0.05)	*0.53*	0.17 − 0.52, 0.86	0.74 (0.05)	*< 0.001*	0.63 0.38, 0.89
	No	0.45 (0.06)			0.58 (0.07)			0.58 (0.07)			0.66 (0.05)			0.6 (0.07)		
I trust parents	Yes	0.76 (0.06)	*0.11*	0.82 − 0.03, 1.66	0.76 (0.03)	*0.003*	1.46 0.69, 2.22	0.72 (0.06)	*0.0004*	0.71 0.37, 1.06	0.74 (0.03)	*0.3*	0.69 − 0.11, 1.49	0.73 (0.05)	*< 0.001*	0.81 0.52, 1.11
	No	0.45 (0.08)			0.5 (0.08)			0.58 (0.08)	*0.006*		0.57 (0.08)			0.57 (0.07)		

*p* < 0.05 indicates moderate statistically significant results, *p* < 0.01 indicates strong evidence of statistically significant results, *p* < 0.001 indicates very strong evidence of statistically significant results

#### Sexuality

Among gay and lesbian participants, those who indicated that they felt that they did not belong at school reported significantly lower HR-QoL, compared with those who felt that they belonged at school (*p* = .02). The effect size, as measured by Cohen’s *d*, was 0.83, indicating a large effect size. On other hand, gay and lesbian participants who felt that they could be themselves at school reported significantly higher HR-QoL scores compared with those who did not, and this was associated with a medium effect size (*g* = 0.75, *p* < .001). Furthermore, gay and lesbian participants who felt that their parents accepted them or that they trusted their parents reported significantly higher HR-QoL scores compared with those who did not feel the same way (*g* = 1.41, *p* < .01; *g* = 1.46, *p* < .003; respectively).

Among bisexual participants, those who found it hard to be accepted at school, felt they did not belong at their school, or wished they were at a different school reported significantly lower HR-QoL scores, compared with bisexual participants did not experience these feelings. A large effect size was observed in relation to the difference in standardised mean HR-QoL scores among bisexual participants who felt that they did not belong at their school compared with bisexual participants who did not (Cohen’s *d* = 0.99, *p* < .001). Inversely, bisexual participants who felt that they can be themselves at school, their friends are good friends, they can trust their friends, their parents accept them, their parents understand them, and they trust their parents reported significantly higher HR-QoL scores compared with those who did not indicate these experiences (Table 4).

No statistically significant differences in weighted standardised means of HR-QoL scores were detected among participants with other diverse sexualities in relation to any of the school-, peer-, and family-level factors examined in this study.

At a group level, sexually diverse people who found it hard to be accepted at school, felt that they did not belong at their school, or wished they were at a different school reported significantly lower HR-QoL scores compared with sexually diverse people who did not report those experiences and feelings (Table 4). The difference in HR-QoL between those who felt that they did not belong at their school compared with those who did produced a large effect size (*g* = − 0.89, *p* < .001). On the other hand, sexually diverse participants who felt they could be themselves at school; felt that their friends are good friends; felt that they could trust their friends; felt that their friends respect their feelings; or indicated parental acceptance, understanding, or trust reported significantly higher HR-QoL scores compared with sexually diverse participants who did not report these experiences or feelings (Table 4).

## Discussion

This study is the first to use population-based, nationally representative data to estimate and track through adolescence the burden and magnitude of HR-QoL and related disparities among LGBTQA2S+ young people in Australia. This study is also the first to quantify the impact of poor mental health through adolescence on HR-QoL among LGBTQA2S+ young people and moreover, estimate differences in HR-QoL among LGBTQA2S+ young people associated with school-, peer-, and family-level factors.

Our findings highlight the scale of HR-QoL inequities affecting LGBTQA2S+ young people, which were well-established at ages 14 and 15 and widened through adolescent years. Moreover, our robust findings highlight that poor mental health likely bears pronounced, deleterious effects on quality of life among LGBTQA2S+ young people through adolescence, a finding of particular importance to policy makers and health system administrators given large-scale, rigorous research consistently highlighting the stark mental health inequities affecting LGBTQA2S+ young people around the world [3, 8]. Our findings provide robust evidence to support the position that school-level and parent-level factors centring inclusivity, acceptance, and a sense of belonging may improve the quality of life of LGBTQA2S+ young people and thus, buffer and avert the quality of life burden associated with health inequities faced by LGBTQA2S+ young people, including mental health challenges.

These findings extend the available literature, which does not include gender and sexuality-based differences [24] or a focus on sexually diverse adults [25, 49]. Strikingly, our study observed that HR-QoL inequities widened most for trans young people, who also reported the most pronounced inequities in HR-QoL at ages 18 and 19, compared to other LGBTQA2S+ subgroups. It is difficult to compare these trans-specific estimates with previous studies due to the small number of trans people included in this study. However, a previous Australian-based study of 525 trans children and adolescents (median age = 14 years) attending a gender-affirming care service reported significantly lower mean HR-QoL scores compared to the LSAC sample reported in this study [23]. Hence, despite the population-based, nationally representative strength of LSAC, this small sample of community-based trans participants may not be representative of all trans adolescents in Australia.

Across the sexually diverse identity subgroups, bisexual participants, and participants with other diverse sexualities experienced disparities in HR-QoL over time relative to their heterosexual peers. It is interesting to note disparities in HR-QoL through adolescence were not detected among gay and lesbian participants. Future prevention efforts should equitably allocate resources to meet the needs of bisexual and broader sexually diverse young people, tailoring mental health promotion messaging to their unique experiences of sexuality as distinct from gay and lesbian participants.

Evidence-based public health policy responses are required to address the dire HR-QoL inequities documented in this study among LGBTQA2S+ young people. Strategic top-down public health documents, including preventive [50] and adolescent-focused [51] policies, should include firm language to direct resources to address these disparities, without shying away from lending credence to LGBTQA2S+ specific interventions and activities, including the provision of safe and timely gender-affirming care for trans young people [23, 52, 53] and cultivation of inclusive school environments for all LGBTQA2S+ young people [54–56]. In accordance with the axioms of public health purported in the Ottawa Charter for Health Promotion [57], public health practitioners should enable LGBTQA2S+ young people to increase control over and improve their health. Flexible responsiveness to the self-determination of LGBTQA2S+ young people is part and parcel of legislating effective policy responses that are sensitive and appropriate to their HR-QoL needs.

Our findings highlight the importance of targeting school, peer, and family settings to improve LGBTQA2S+ young peoples’ health and well-being. Particularly, positive improvements in HR-QoL were consistently associated with school-level factors, including promoting feelings of acceptance and belonging and freedom of self-expression. Our findings provide support for future investment in school initiatives such as gender and sexuality alliances, anti-LGBTQA2S+ bullying policies, inclusive schooling curriculum, and staff and student training and education on gender and sexuality diversity, which effectively promote inclusivity and acceptance of LGBTQA2S+ young people [30]. School-based efforts to promote the health and well-being of LGBTQA2S+ young people are especially appealing as they may confer benefits that extend beyond the school settings to peer-level [58] and family-level contexts [29]. For example, there are available LGBTQA2S+-specific school-based resources that promote the capacity of LGBTQA2S+ young people to advocate, communicate, trust, and establish allyship with their families which in turn, provide academic, emotional, and prosocial benefits in classrooms [29, 59, 60]. Hence, given the multidimensional benefits of school-based preventive health efforts for the health and well-being of LGBTQA2S+ young people, this study provides robust health evidence to support efficient investment in school-based strategies that aim to foster feelings of LGBTQA2S+ acceptance, belonging, freedom of expression, and community. Conversely, future investment is warranted in parent- and family-based interventions that seek to depathologise experiences of LGBTQA2S+ young people, providing evidence-based information and LGBTQAS+ community connections to parents and families to facilitate acceptance and support for their LGBTQA2S+ children [61–63].

Future research should employ additional health economic analytic methodologies to quantify the economic costs associated with the burden of HR-QoL and mental health inequities affecting LGBTQA2S+ through adolescence. Future research should also quantify the cost savings and benefits associated with the aversion, reduction, and prevention of these disparities through the employment of school, peer, and family-based interventions promoting acceptance, inclusion, and support for LGBTQA2S+ growing up queer and/or trans in Australia. Accordingly, future research efforts should adopt a life-course perspective, examining the impacts of HR-QoL and mental ill-health inequities impacting LGBTQA2S+ young people, across the life-span.

This study is not without limitations. Although this study leveraged longitudinal, population-level nationally representative data from a probability sample, this study used the CHU-9D [37] to measure HR-QoL. A recent study of university students aged 18 to 29 years produced cautionary findings regarding the potential use of the CHU-9D for individuals aged 18 to 19 years, given research consistently showing that adolescents tend to produce lower health-state values than adult values [64]. However, given the researchers were focused on the preferences of adolescents for the present cost-effectiveness analyses to inform future treatment and service outcomes that are more relevant to LGBTQA2S+ young peoples’ preventive health needs [64], it was justified to proceed with CHU-9D use, particularly given the longitudinal nature of this study, which focused on the same cohort of adolescents through their developmental years while controlling for their within-subject error variation. Another relevant limitation is that the present study did not correct for multiple statistical comparisons, hence some statistically significant findings may be artificial due to an inflated false positive rate (type I error). Lastly, it should be noted that the items used to measure gender and sexuality in the LSAC study had significant limitations and may have failed to capture the experiences of nonbinary, gender diverse, and agender young people and those attracted to others regardless of gender. Future research should utilise population-level studies as an invaluable opportunity to measure the breadth of experiences of gender and sexuality diversity among young people.

Relative to their cisgender, heterosexual peers, LGBTQA2S+ young people experience significant disparities in HR-QoL in early adolescence that widen through adolescence, especially among trans young people. Policymakers and public health practitioners have a timely opportunity to address these vast health inequities affecting LGBTQA2S+ young people through strategic policy agenda setting and the cost-effective delivery of evidence-based preventive health activities, particularly in school settings.

For too long, research efforts have repeatedly drawn attention to the magnitude of mental health disparities experienced by LGBTQA2S+ young people in a deficit-focused manner. Our findings highlight that it is time for change, making the case for policy makers and programme evaluators to prioritise addressing the inequities in quality of life experienced by LGBTQA2S+ young people by targeting school-level and parent-level factors associated with fostering inclusivity, acceptance, and a sense of belonging from early adolescence.
